# Gregatins, a Group of Related Fungal Secondary Metabolites, Inhibit Aspects of Quorum Sensing in Gram-Negative Bacteria

**DOI:** 10.3389/fmicb.2022.934235

**Published:** 2022-07-05

**Authors:** Wouter A. G. Beenker, Jelmer Hoeksma, Jeroen den Hertog

**Affiliations:** ^1^Hubrecht Institute-KNAW and University Medical Center Utrecht, Utrecht, Netherlands; ^2^Institute Biology Leiden, Leiden University, Leiden, Netherlands

**Keywords:** quorum sensing, gregatins, *Chromobacterium violaceum*, *Pseudomonas aeruginosa*, antimicrobial activity, fungi

## Abstract

Quorum sensing (QS) is a process that regulates gene expression based on cell density. In bacteria, QS facilitates collaboration and controls a large number of pathways, including biofilm formation and virulence factor production, which lead to lower sensitivity to antibiotics and higher toxicity in the host, respectively. Inhibition of QS is a promising strategy to combat bacterial infections. In this study, we tested the potential of secondary metabolites from fungi to inhibit bacterial QS using a library derived from more than ten thousand different fungal strains. We used the reporter bacterium, *Chromobacterium violaceum*, and identified 39 fungal strains that produced QS inhibitor activity. These strains expressed two QS inhibitors that had been described before and eight QS inhibitors that had not been described before. Further testing for QS inhibitor activity against the opportunistic pathogen *Pseudomonas aeruginosa* led to the identification of gregatins as an interesting family of compounds with QS inhibitor activity. Although various gregatins inhibited QS in *P. aeruginosa*, these gregatins did not inhibit virulence factor production and biofilm formation. We conclude that gregatins inhibit some, but not all aspects of QS.

## Introduction

Antibiotic resistance is a growing problem leading to ineffective antibiotic treatments, causing bacterial infections to be lethal (Falagas and Bliziotis, 2007; Laxminarayan et al., 2016; Cassini et al., 2019; Ventola, 2019). In particular, the treatment of Gram-negative bacteria is challenging due to the composition of their outer membrane, which makes it hard for antibiotics to enter the cells (Zgurskaya et al., 2015; Masi et al., 2017; Richter and Hergenrother, 2018). While new antibiotics are still being introduced into the clinic, these often represent synthetically optimized antibiotics from existing classes, leading to a quick rise in resistance (Fischbach and Walsh, 2009). Therefore, it is important to look for alternative approaches to fight bacterial infections. Targeting bacterial quorum sensing (QS) is one of these promising approaches.

Quorum sensing is an effective bacterial communication system that is triggered by changes in cell density. Bacteria secrete signal compounds, termed autoinducers. In the case of Gram-negative bacteria, these autoinducers are acyl-homoserine lactones (AHLs) produced by LuxI-type autoinducer synthases. The AHLs cross the membrane and bind to LuxR-type receptors. If the cell population density increases, the signal increases, eventually leading to altered gene expression (Bassler and Losick, 2006). QS regulates various pathways involved in the production of virulence factors and strengthening of the biofilm (Passador et al., 1993; Jakobsen et al., 2017; Defoirdt, 2018). The opportunistic pathogen *Pseudomonas aeruginosa* has three different QS pathways: two N-acyl homoserine lactone (AHL)-based pathways (*las*-encoded system and *rhl*-encoded system) and a unique *Pseudomonas* quinolone signal (*pqs*)-based pathway. These three pathways are interconnected through feedback and feed-forward mechanisms (Lee and Zhang, 2014; Mukherjee et al., 2018). Some studies refer to IQS as the fourth QS system in *P. aeruginosa*, but because this is controversial (Cornelis, 2020), we have not addressed IQS here. The QS system in *P. aeruginosa* plays a role in the production of virulence factors, including elastase (Passador et al., 1993), protease, (Gambello et al., 1993) and pyocyanin (Brint and Ohman, 1995), and strengthens biofilm formation by the production of rhamnolipids (Ochsner and Reiser, 1995) and extracellular DNA (Allesen-Holm et al., 2006; Jakobsen et al., 2017). In general, inhibition of QS decreases the production of toxic virulence factors and weakens biofilm formation (Bjarnsholt et al., 2005; Jakobsen et al., 2012). Therefore, inhibition of QS may have beneficial effects, including less tissue damage, due to reduced levels of toxic virulence factors and higher susceptibility to antibiotics, because of the weakened biofilm. QS inhibitors have been isolated from various sources over the years, including ajoene from garlic (Jakobsen et al., 2012), quercetin from oak (Gopu et al., 2015), furanones from alga (Givskov et al., 1996), and flavones from natural origin or synthetically generated flavones (Skogman et al., 2016).

In general, fungi are an interesting source of natural compounds that have progressed into the clinic (Brakhage, 2013; Keller, 2018). For instance, the antibiotics penicillin and cephalosporin have a fungal origin and have been used to treat many patients (Brakhage, 2013). Yet, fungi remain rather unexplored with respect to the production of QS inhibitors (Kalia, 2013). Nevertheless, QS inhibitors have also been found to be produced by fungi, including patulin and penicillic acid (Rasmussen et al., 2005b), making them an interesting potential source for QS inhibitors. In collaboration with the Westerdijk Fungal Biodiversity Institute, our lab has developed a unique library that consists of filtrates of 10,207 fungal strains (Hoeksma et al., 2019), which facilitates the search for novel natural compounds produced by a large variety of fungal species.

The aim of this study was to identify novel QS inhibitors. To this end, we screened our library of fungal filtrates, which allowed us to assess the potential of QS inhibition among 10,207 strains of fungi. For the screening, we used the Gram-negative bacterium *Chromobacterium violaceum* as a reporter. *C. violaceum* produces violacein, a purple pigment, upon activation of QS, making it an excellent reporter for high-throughput screens (Skogman et al., 2016; Manner and Fallarero, 2018). This approach led to the identification of eight compounds with QS inhibitor activity that had not been described before. In addition, we tested selected compounds for inhibition of specific aspects of QS in the opportunistic pathogen *Pseudomonas aeruginosa*. We identified gregatins as a promising group of compounds to inhibit QS in various Gram-negative bacterial strains.

## Materials and Methods

### Bacterial Strains and Growth Conditions

The bacterial strains used in this study are listed in Table 1. Bacteria were stored at −80°C in a 20% glycerol stock solution. *C. violaceum* was inoculated on tryptic soy agar (TSA), and single colonies were grown in tryptic soy broth (TSB) at 27°C. PAO1 strains were inoculated on Luria agar (LA) at 37°C, and single colonies were grown in AB minimal medium supplemented with 0.5% glucose and 0.5% casamino acids (Jakobsen et al., 2018), unless stated otherwise.

**Table 1:** Bacterial strains used in this study.

**Bacterial strain**	**Characteristic**	**Source**
*Chromobacterium violaceum*	WT, ATCC 12472	Westerdijk Fungal Biodiversity Institute
*P. aeruginosa*	WT, PAO1	
*PAO1-GFP*	WT, PAO1 *gfp*-tagged	Yang et al. (2007)
*PAO1 lasB-GFP*	WT, PAO1, *gfp* fusion to *lasB* gene	Hentzer et al. (2002)
*PAO1 rhlA-GFP*	WT, PAO1, *gfp* fusion to *rhlA* gene	Fong et al. (2017)
*PAO1 pqsA-GFP*	WT, PAO1, *gfp* fusion to *pqsA* gene	Fong et al. (2017)
*PAO1 ΔlasI-ΔrhlI*	PAO1, QS mutant	Hentzer et al. (2003)

### High-Throughput Screen for Quorum-Sensing Inhibitors

The high-throughput screen for QS inhibitors using *C. violaceum* as a reporter was performed as previously described, with minor modifications (Skogman et al., 2016; Manner and Fallarero, 2018). Overnight grown *C. violaceum* was diluted and grown until an OD_600_ of 0.5–0.7. Then, the bacteria were diluted 1:1,000 before addition to the 96-well plate containing the fungal supernatant to a total volume of 80 μl (1:1, v:v). In addition to the wells with fungal supernatant, plates also included untreated bacteria and TSB only to check the sterility of the medium. Quercetin (Sigma-Aldrich, Merck Life Science, Amsterdam, the Netherlands) was added at a concentration of 125 μM as a positive control of violacein inhibition, and 130 μM of meropenem (Sigma-Aldrich, Merck Life Science, Amsterdam, the Netherlands) was used as a control in the viability assay. Plates were incubated for 20 h at 27°C with 200 rpm shaking.

To measure the violacein production, plates were centrifuged at 3,000 rpm for 10 min to collect the precipitated violacein. The supernatant was discarded, and the pellet was resuspended in 200 μl of 96% ethanol. Plates were centrifuged again at 3,000 rpm for 10 min to separate the cells from the violacein to avoid interference with the signal. Half of the supernatant was transferred to a new 96-well plate, and the violacein production was quantified by measuring the optical density at 562 nm on the ASYS expert plus microplate reader (Biochrom Ltd, Cambridge, UK).

Resazurin staining was used to measure the viability of the bacteria. Following incubation for 20 h as described earlier, plates were centrifuged at 3,000 rpm for 10 min. The supernatant was removed, and resazurin (25 μg/mL in PBS) solution was added. Plates were incubated in darkness for 45 min at 27°C before fluorescence was measured on a PHERAstar microplate reader (BMG Labtech, de Meern, the Netherlands) using 540 nm excitation and 590 nm emission wavelength. Viability was calculated using the following equation:


$$\begin{array}{l} \%\;Viability\;=\;\frac{Fluorescence\;sample\;-\;Fluorescence\;background}{Fluorescence\;untreated-Fluorescence\;background} \end{array}$$

The same approach was used in the follow-up experiments, which measured the effect of single molecules on violacein production. Dilution ranges of the compounds were tested in triplicates using a maximum concentration of 2.5% DMSO to minimize the effect of DMSO on QS and bacterial viability.

### QS Inhibition in *Pseudomonas aeruginosa*

The experiments were performed as previously described (Jakobsen et al., 2018). In brief, overnight grown cultures were diluted in PBS to an OD_450_ of 0.1–0.2 before addition to a 96-well plate with dilution ranges of compounds up to a volume of 200 μl (1:1, v:v). The GFP fluorescence (excitation 485 nm, emission 535 nm) and absorbance (600 nm) were measured every 15 min for 15 h at 34°C on a CLARIOstar microplate reader (BMG Labtech, de Meern, the Netherlands). IC_50_ values were calculated using PRISM software, by plotting the maximum slope of GFP/OD.

### Biofilm Assay

Overnight cultures were diluted 1:1,000 to a final OD_600_ of ~0.01. Diluted bacterial cells were added to a 96-well plate containing concentration ranges of the compound in triplicates up to a volume of 200 μL (1:1, v:v). Plates were sealed with BreatheEasy seal (Sigma-Aldrich, Merck Life Science, Amsterdam, the Netherlands) to prevent evaporation and incubated at 37°C under static conditions. After 24 h, the medium was discarded, and the wells were rinsed once with PBS. The biomass was then stained with 0.1% (w:v) crystal violet solution for 5 min. Crystal violet was discarded, and excess crystal violet was removed by rinsing three times with water. Plates were dried overnight, and bound crystal violet was resuspended in 33% (v:v) acetic acid and quantified at a wavelength of 562 nm using an ASYS expert plus microplate reader (Biochrom Ltd, Cambridge, UK).

### Pyocyanin Extraction

Pyocyanin quantification was based on a previously described assay with minor modifications (Essar et al., 1990). In brief, treated bacteria were grown in Kings A medium (2% (w:v) protease peptone, 1% (w:v) potassium sulfate, 0.164% (w:v) magnesium chloride, and 1% (v:v) glycerol in MQ) for 24 h at 37°C in triplicates, before pelleting the cells. About 900 μL of bacterial supernatant was added to chloroform (1:1) and tubes were shaken vigorously. Then, 800 μL of chloroform was added to 700 μL of 0.2 M HCl and vortexed. Samples were centrifuged for 2 min at 10,000 rpm, and 600 μL of 0.2 M HCl was transferred to a cuvette. Absorbance was measured at 520 nm, using 0.2 M HCl as a blank.

### Rhamnolipid Extraction

Rhamnolipid concentrations were measured based on the standard orcinol-sulfuric acid assay (Zhou et al., 2019). In brief, treated cultures were grown at 37°C for 24 h before collecting 900 μL of supernatant. Diethyl ether was added (1:1) to the supernatant and mixed. Then, 800 μL of diethyl ether was taken to a fresh tube and dried *via* evaporation at RT. To each extract, 100 μL of MQ was added before the addition of another 800 μL of 12.9 mM orcinol (Sigma-Aldrich, Merck Life Science, Amsterdam, the Netherlands) in 70% (v:v) H_2_SO_4_. The reaction was maintained at 80°C for 30 min, and absorbance was measured at a wavelength of 495 nm.

### Purification of Compounds

Fungal strains corresponding to the active filtrates were grown on a specific agar plate preferred by the strain and incubated at 25°C. After 7 d, cubes of 5 × 5 mm were cut out and two cubes were used per 50 mL of medium in 100 mL bottles. Standard medium consisted of 3.5% Czapek dox broth + 0.5% yeast extract. To produce gregatins, potato dextrose broth (23% (v:v) potato extract + 2% glucose) was used. Fungi were incubated in a liquid medium for 7 d at preferred conditions (15°C static, 25°C static, or 25°C + 100 rpm on an orbital shaker) before filter sterilizing the medium with a 0.22 μm Millipore filter (Merck, Amsterdam, the Netherlands). The sterile supernatant was extracted using 3 × 1/3 volume of ethyl acetate using a separation funnel. The ethyl acetate layers were collected and evaporated to dryness using a rotary evaporator with a water bath at 40°C. The dried pellet was dissolved in DMSO. The extracts were fractionated using a preparative high-performance liquid chromatography (HPLC) system consisting of a Shimadzu CBM-20A controller, a Shimadzu LC-20AP pump, and a Shimadzu FRC-10A fraction collector using a C18 reversed-phase Reprosil column (10 μm, 120 Å, 250 × 22 mm) and a Shimadzu SPD-20A UV-detector set at 214 and 254 nm (Shimadzu's Hertogenbosch, the Netherlands). The mobile phase consisted of 100% MQ with 0.1% trifluoroacetic acid (buffer A) and 100% acetonitrile with 0.1% trifluoroacetic acid (buffer B). Protocols consisted of the following steps: 5% buffer B for 5 min, followed by a linear gradient to 95% buffer B for 40 min, 5 min of 95% buffer B before returning to 5% buffer B for another 5 min with a constant flow rate of 12 mL/min. Fractions were collected every 63 s starting after the DMSO peak and ending at 95% buffer B, resulting in 40 fractions. About 1.9 mL of the fraction was dried in a speed-vac overnight and dissolved in 50 μl of DMSO to test for QS inhibitory activity.

### Identification of Compounds

Active fractions were analyzed for their purity using an analytical Shimadzu LC-2030 system with PDA detection (190–800 nm) with a Shimadzu Shim-pack GIST C18-HP reversed-phase column (3 μm, 4.6x100 mm) (Shimadzu's Hertogenbosch, the Netherlands). Besides determining the purity, the UV-VIS spectrum of the fractions was also obtained. Pure fractions were further analyzed by measuring the mass using a Shimadzu LC-2030C 3D plus system, sometimes followed by more accurate high-resolution mass spectrometry (HRMS) measured on an LCT instrument (MicroMass ltd, Manchester UK). For HRMS, the sample was mixed with sodium formate for the detection of sodium adduct ions. In addition, this procedure gave an internal calibrant to each sample to facilitate a more accurate measurement of the mass of the sample. Obtained masses and UV spectra were compared with previously identified samples and literature. If needed, further chemical analysis using NMR measurements was performed. For the NMR measurements, the fractions were dried in a speed-vac overnight and dissolved in DMSO-d_6_ before measurements on a Bruker 600 MHz.

### Commercial Compounds Used

Rubrofusarin (Sigma-Aldrich) was used to test for QS inhibitory activity. Penicillic acid (VWR) and indole-3-acetic acid (Thermo-Fisher) were used to compare with identified fungal compounds.

## Results

### Screen for Quorum-Sensing Inhibitors

To search for novel QS inhibitors, we used a high-throughput method using a Gram-negative bacterium, *C. violaceum*, as a reporter strain, which produces a purple pigment violacein upon activation of QS (Skogman et al., 2016; Manner and Fallarero, 2018). As a source of potential QS inhibitors, we used a library consisting of the secondary metabolites of 10,207 fungal strains, which was described by Hoeksma and colleagues (Hoeksma et al., 2019). All fungi were obtained from the Westerdijk Fungal Biodiversity Institute. The fungi we selected for further analysis are depicted in Table 2. The fungal supernatant was added (1:1, v:v) to *C. violaceum*, and after overnight incubation, the amount of violacein was determined.

**Table 2:** Compounds with QS inhibitor activity.

**Compound**	**IC_**50**_ Violacein**	**IC_**50**_** **viability**	**Fungus**
Penicillic acid	22.13 μM	126.2 μM	*Aspergillus auricomus (CBS639.78)*
			*Aspergillus melleus (CBS622.75)*
			*Aspergillus ostianus (CBS627.78)*
			*Aspergillus sulphureus (CBS117.26)*
			*Eupenicillium baarnense (CBS315.59)*
			*Penicillium simplicissimum (CBS392.78A)*
			*Penicillium simplicissimum (CBS391.78A)*
Patulin	12 μM	22 μM	*Metarhizium brunneum (CBS316.51)*
			*Penicillium tardum (CBS378.48)*
Indole-3-acetic acid	481 μM	6091 μM	*Colletotrichum fragariae (CBS142.31)*
6-methyl salicylic acid	419 μM	5789 μM	*Penicillium tardum (CBS378.48)*
Citrinin	201 μM	~1-2 mM	*Aspergillus allahabadii (CBS164.63)*
			*Penicillium citrinum (CBS309.48)*
			*Penicillium citrinum (CBS252.55)*
			*Penicillium citrinum (CBS341.61)*
			*Penicillium citrinum (CBS139.45)*
			*Penicillium spinulosum (CBS294.62)*
Rubrofusarin	92 μM	>250 μM	*Commercial compound*
desmethyl gregatin A	14 μM	>1 mM	*Aspergillus allahabadii (CBS164.63)*
Gregatin A	344 μM	>4 mM	*Aspergillus panamensis (CBS120.45)*
Gregatin D	210 μM	>4 mM	*Aspergillus panamensis (CBS120.45)*
Cyclogregatin	26 μM	>1 mM	*Aspergillus panamensis (CBS120.45)*

*Compounds with QS inhibitor activity are listed. IC_50_ for QS inhibitor activity (violacein production) and IC_50_ for viability of C. violaceum bacteria are depicted with the name of the fungi that produce the respective compounds*.

**Figure 1:**
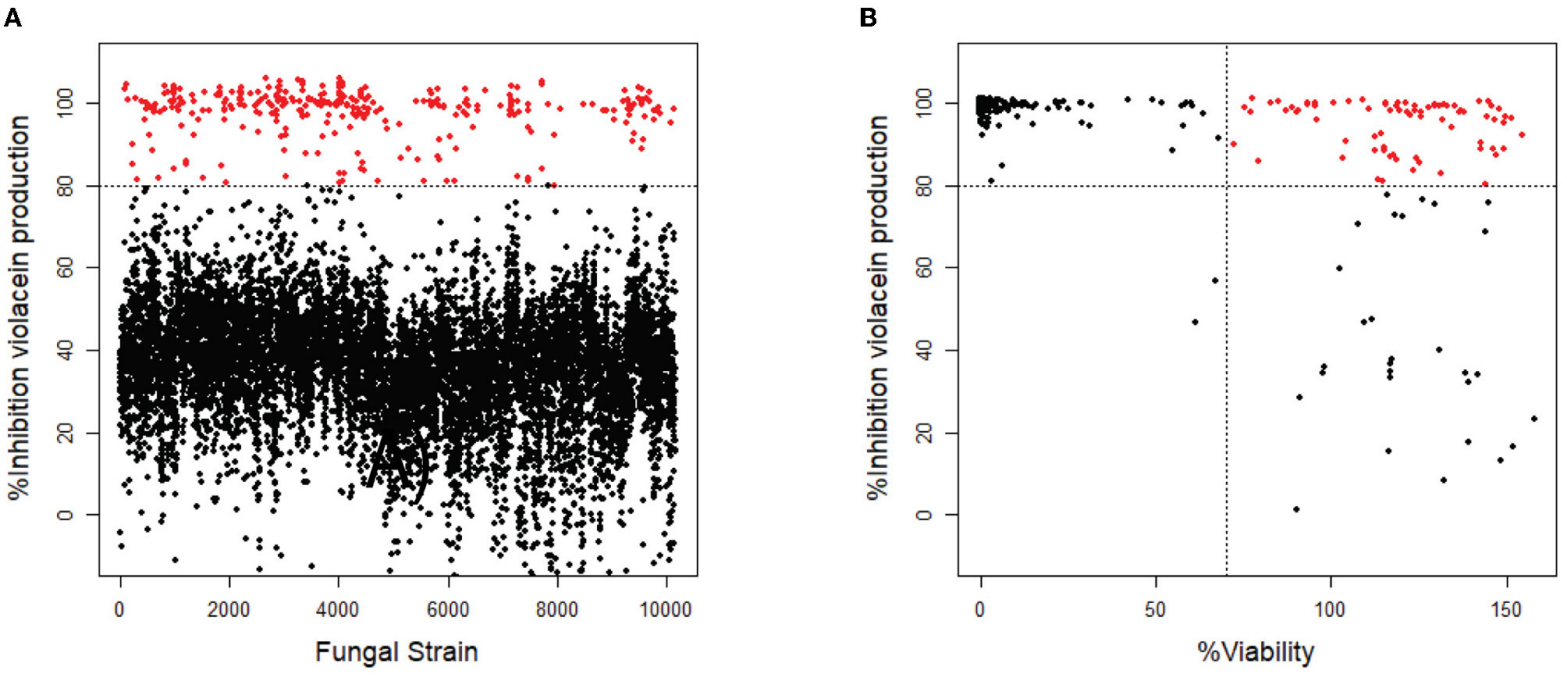
Screening of fungal secondary metabolites on *C. violaceum* reporter. **(A)** Inhibition of violacein production after treatment with fungal supernatant. Every dot represents the supernatant of a single fungal strain. Fungal supernatants that show inhibition of more than 80% are shown as red dots (*N* = 324). **(B)** The 324 strains that showed inhibition of >80% were screened again, measuring both inhibition of violacein production and the viability of the bacteria. The compounds that show inhibition of >80% while not affecting the viability (>70%) are shown as red dots (*N* = 79).

Of the 10,207 fungal filtrates, 324 inhibited violacein production by more than 80% when compared to untreated (Figure 1A). Since the loss of violacein production might also be due to the loss of viability of the bacteria, analysis of violacein production in response to the 324 hits was repeated, and the viability of the bacteria was determined in parallel using a resazurin staining assay (Guerin et al., 2001; Rampersad, 2012). Seventy-nine strains were identified to inhibit violacein production without affecting the viability. Viability is calculated as the ratio of fluorescence intensity in the sample and fluorescence intensity in the control. Violacein interferes somewhat with fluorescence. Therefore, it is not surprising that in samples with high QS inhibitor activity and hence low violacein production, the apparent fluorescence in the resazurin assay is higher than in the control and thus higher than 100%. A total of 214 strains reduced violacein production and at the same time reduced the viability of *C. violaceum*, which presumably caused the observed reduction in violacein production. Thirty-one strains from the initial screen did not affect violacein production significantly and hence, turned out to be false positives (Figure 1B).

The 79 strains that inhibited violacein production without affecting the viability of *C. violaceum* were selected for further analysis. The fungi were cultured again, and the growth conditions were optimized (15°C static, 25°C static, and 25°C with shaking) to maximize the inhibitory response. Of the 79 hits, 39 showed activity after reculturing: seven preferred 15°C cultivation, 20 preferred 25°C cultivation, and 12 preferred 25°C cultivation with shaking. Of the other strains that were cultured again, 29 did not show QS inhibitor activity in any of the growth conditions, and 11 turned out to be toxic for the bacteria. These 40 strains were disregarded. The remaining 39 fungal strains harbored QS inhibitor activity and were cultured in larger volumes at the optimized growth condition for activity-guided purification of the QS inhibitors and further chemical analysis.

### Identification of Bioactive Compounds

**Figure 2:**
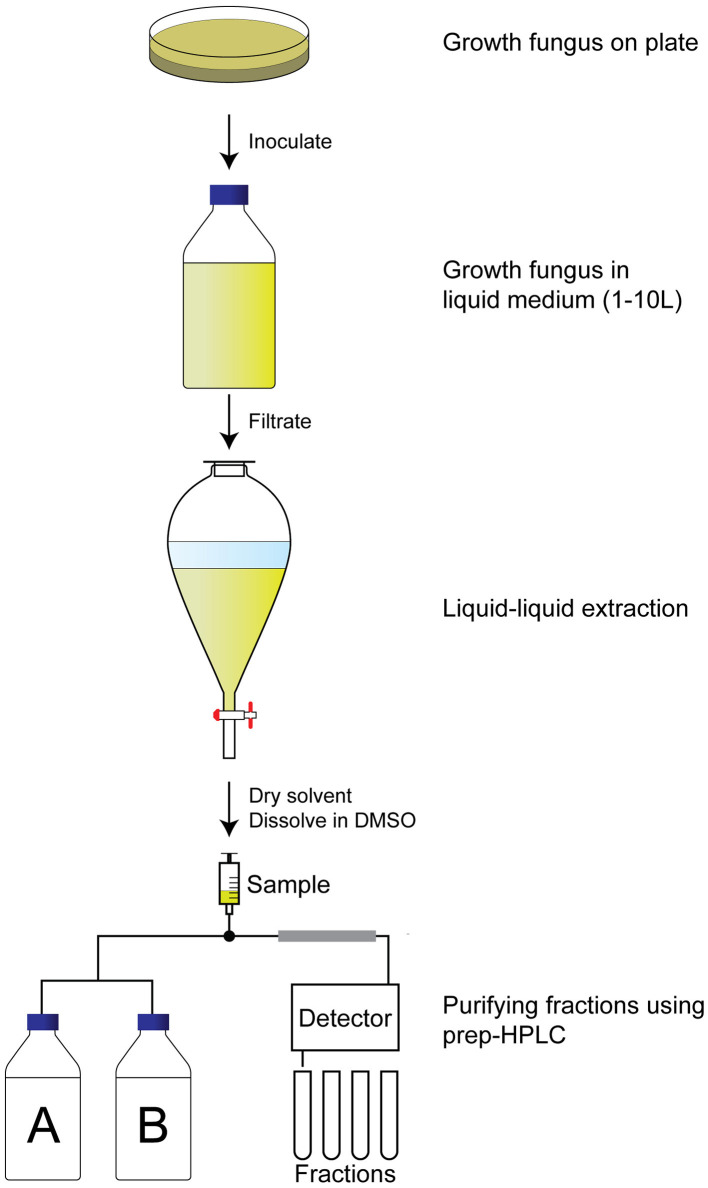
Activity-guided purification approach to identify QS inhibitors. A fungus of interest is grown on an agar plate for a week before inoculation in large volume (1–10 L) for another week under preferred conditions. The supernatant is then filtrated before liquid–liquid extraction with ethyl acetate. The ethyl acetate is dried, and the pellet is dissolved in DMSO before fractionating the extract using a preparative HPLC. Fractions are dried and tested for QS inhibitory activity before identification of the fraction.

To identify the compounds with QS inhibitor activity, an activity-guided purification approach was used (Figure 2). Briefly, after 7 d of growth in the liquid medium, the supernatant was separated from the fungus by filtration. The secondary metabolites were isolated by liquid–liquid extraction, evaporation of the solvent, and dissolving in DMSO. The samples were tested for QS inhibitor activity using violacein production as a read-out. Active samples were analyzed using analytical HPLC, which allowed us to avoid repeated re-identification of the same compounds with identical retention times and UV-VIS absorbance. In case the extract did not appear to contain known compounds with QS inhibitor activity, the extract was fractionated using preparative HPLC, and single fractions were tested for QS inhibitor activity.

The purity of active fractions was examined using analytical HPLC. Pure fractions with QS inhibitor activity were further analyzed using various methods to identify the compound, including LC-MS, high-resolution MS, and ^1^H and ^13^C nuclear magnetic resonance (NMR). If necessary, subsequent 2D-NMR methods were used to identify the chemical structure of the compound, including correlation spectroscopy (COSY), heteronuclear single-quantum correlation (HSQC), and heteronuclear multiple bond correlation spectroscopy (HMBC).

### Proof of Principle: Identification of Penicillic Acid as QS Inhibitor

**Figure 3:**
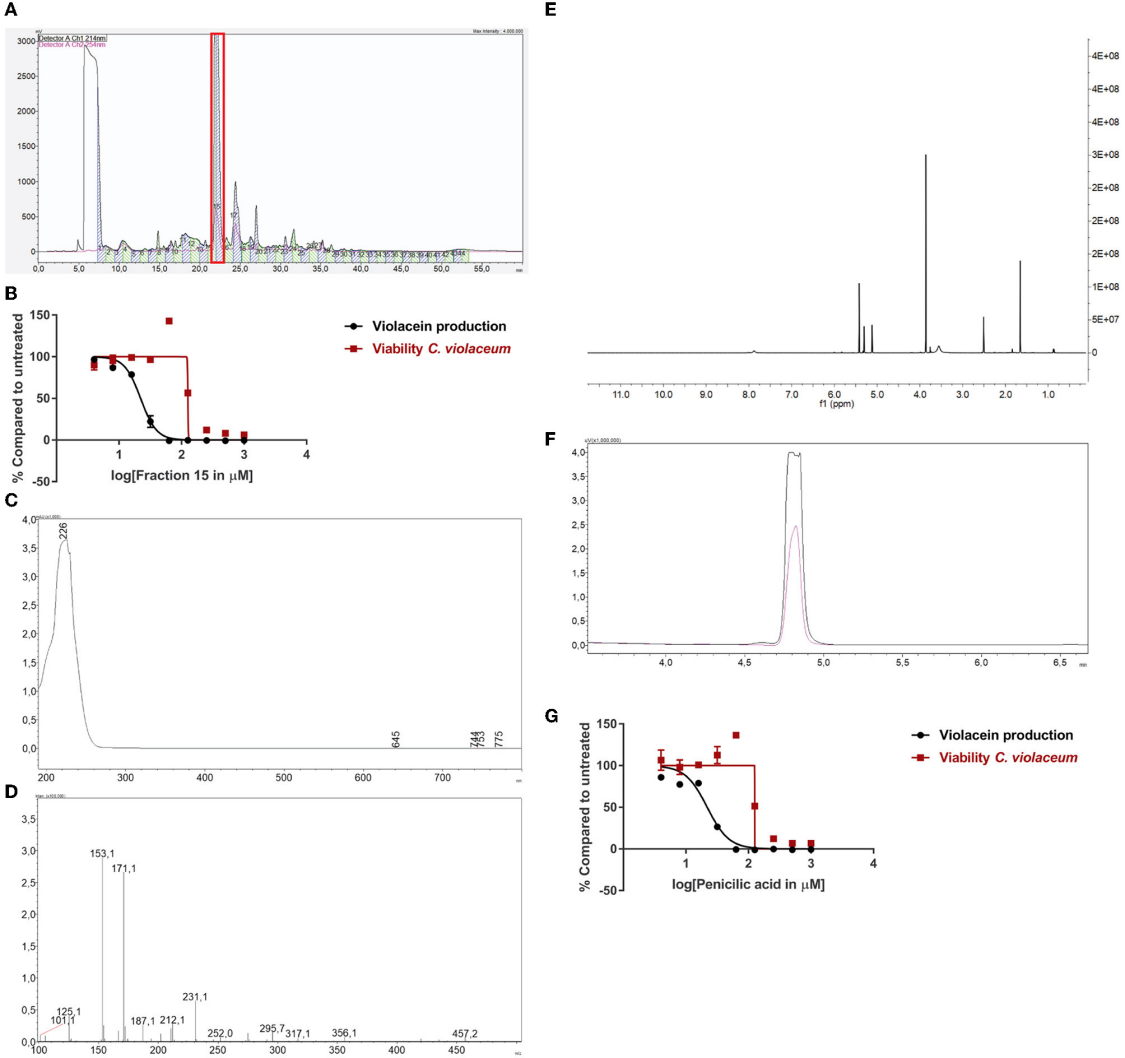
Identification of penicillic acid as QS inhibitor. **(A)** After growing *Penicillium simplicissimum* in large volume, the extract was fractionated using prep-HPLC. Fraction 15 (red box) was the active fraction. **(B)** QS inhibitory activity of fraction 15 using *C. violaceum* as a reporter (black line and symbols) and cell viability in parallel (red line and symbols). A range of concentrations was tested. **(C)** UV-VIS spectrum of fraction 15. **(D)** LC/MS analysis of fraction 15. **(E)** ^1^H-NMR of fraction 15. **(F)** Analytical HPLC chromatogram of fraction 15 (black line) compared to commercial penicillic acid (pink line), showing a similar retention time. **(G)** QS inhibitor activity and viability of commercial penicillic acid. Experiments were done in triplicate. Error bars represent SEM.

One of the active fungi identified during the screening process is *Penicillium simplicissimum* (CBS 392.78A) with a 100% inhibition of violacein production, while viability was not significantly affected (85% compared to untreated). We inoculated this fungus in a large volume at 25°C, extracted the supernatant with ethyl acetate, and dissolved the sample in DMSO. Purification and subsequent analysis of the fractions led us to select fraction 15 as the active fraction (Figure 3A). Sub-lethal levels of this compound show a concentration-dependent violacein inhibition (Figure 3B). Viability levels appear to be upregulated when QS is inhibited. This is likely due to low violacein production at these concentrations, compared to the control, which leads to an enhanced ratio of fluorescence in this assay.

Measuring the bioactive fraction 15 on the analytical HPLC showed a single peak with a maximum UV absorbance of 226 nm (Figure 3C). Further chemical analysis showed an m/z of 171.1 [M + H] (Figure 3D). Next, the fraction was dried and dissolved in DMSO-d_6_ for ^1^H-NMR spectrum analysis (Figure 3E). Analysis of these data suggested that fraction 15 from *Penicillium simplicissimum* contained penicillic acid.

To verify the identity of the active compound in fraction 15, commercially available penicillic acid was analyzed by analytical HPLC (Figure 3F) and tested on *C. violaceum* (Figure 3G). The results showed that the retention time, absorbance, and QS inhibitor activity of commercially available penicillic acid matched that of bioactive fraction 15. Penicillic acid was identified as a QS inhibitor before (Rasmussen et al., 2005a). Our results, together with the published data on penicillic acid, provide proof-of-principle for our approach to identify QS inhibitors.

### Other QS Inhibitors From Fungi

**Figure 4:**
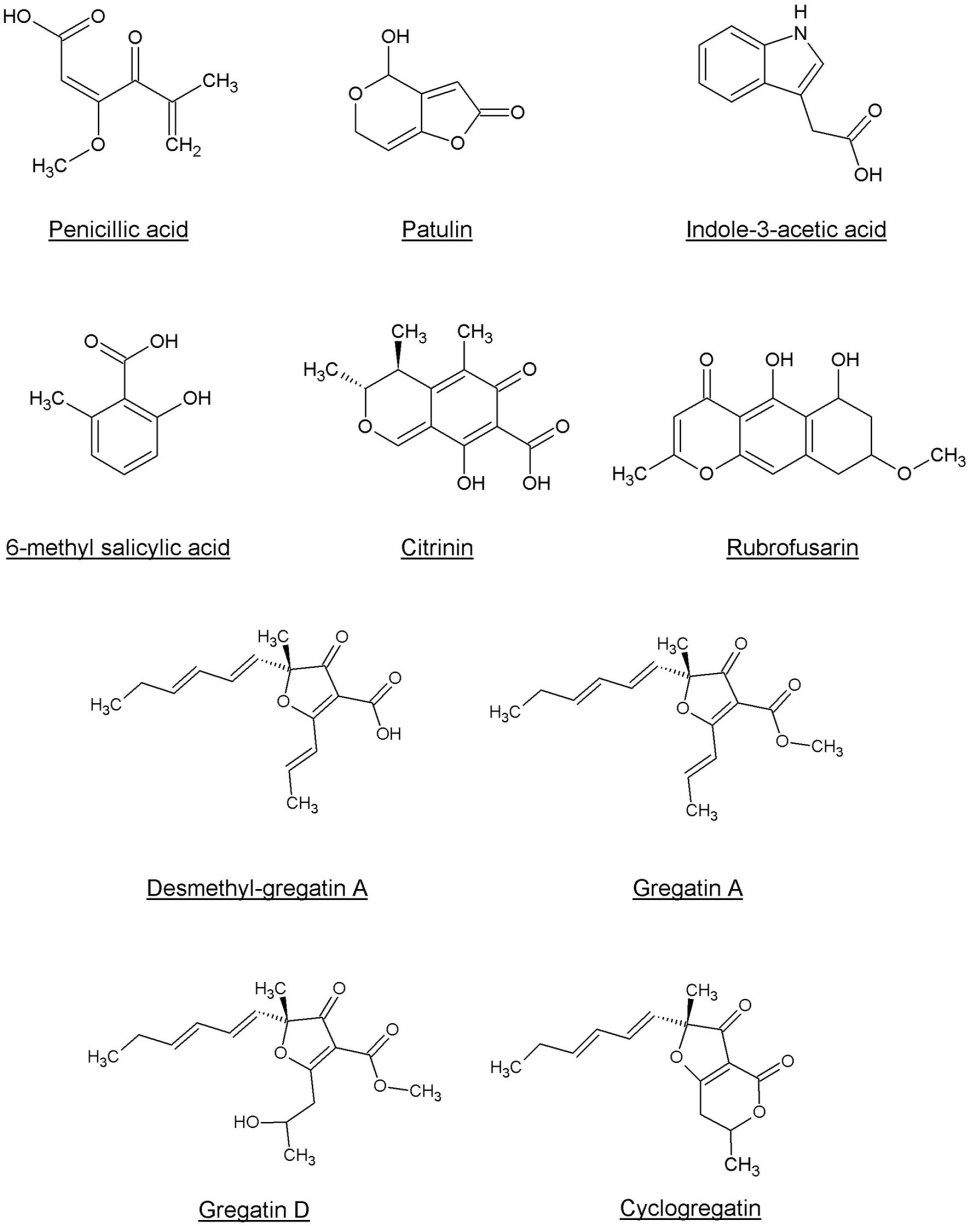
Chemical structures of the compounds identified as QS inhibitors in this study.

Penicillic acid was identified as the bioactive compound in six more fungi, based on analytical HPLC retention time and UV-VIS spectrum (Table 2). Our approach led to the identification of a variety of other known QS inhibitors, including patulin (Rasmussen et al., 2005a) and derivatives or compounds closely related to known QS inhibitors, including 6-methylsalicylic acid and indole-3-acetic acid (Lee et al., 2009; Yang et al., 2009; Tan et al., 2013; Hidalgo-Romano et al., 2014; Monte et al., 2014; Biswas et al., 2015; Ahmed et al., 2019). Interestingly, our approach led to the identification of compounds that had not been described before as QS inhibitors, including citrinin, rubrofusarin, and the family of gregatins (Table 2, Figure 4, Supplementary Figure 1).

Rubrofusarin was detected in the active fraction of *Aspergillus carbonarius* (CBS 101.14). However, the active fraction was not pure and also contained fonsecin (Supplementary Figure 2). We were not able to separate rubrofusarin and fonsecin by preparative HPLC. We obtained a commercially available rubrofusarin and established that it exhibited potent QS inhibitor activity without affecting viability (Table 2, Supplementary Figure 1). Unfortunately, fonsecin is not commercially available, and hence, it remains to be determined if fonsecin also has QS inhibitor activity.

The difference between concentrations that elicited bacterial toxicity and QS inhibitory activity in *C. violaceum* was 2-fold for patulin and 6-fold for penicillic acid, respectively (Figure 3G, Table 2, Supplementary Figure 1). Interestingly, this difference in concentrations was much bigger for the newly identified QS inhibitors. For instance, the IC_50_ of desmethyl-gregatin A, isolated from *Aspergillus allahabadii* (CBS164.63), for the viability of bacteria was 74 times higher than the IC_50_ for violacein inhibition (Table 2, Supplementary Figure 1). These results suggest that these newly discovered QS inhibitors were effective at concentrations that did not affect bacterial viability, and therefore these QS inhibitors were selected for further analysis using other bacterial species.

### QS Inhibitor Activity on *P. aeruginosa*

**Figure 5:**
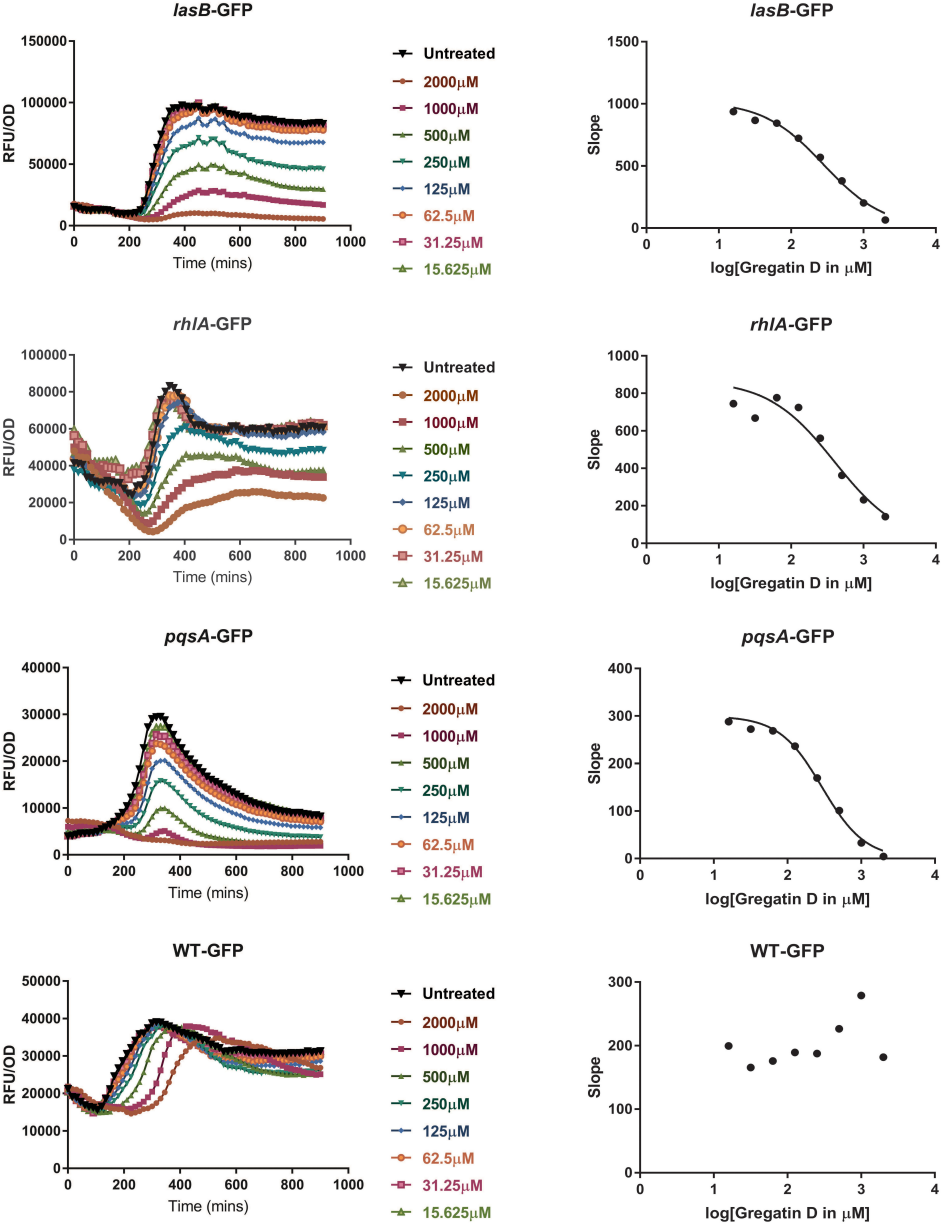
QS inhibitor activity of gregatin D on *Pseudomonas aeruginosa* strain PAO1. Gregatin D was tested on the three QS reporters of *P. aeruginosa* and a WT-GFP control. The GFP signal was normalized by dividing the OD_600_ values giving RFU/OD plots (graphs on the left). The maximum slopes are plotted (graphs on the right) by which the IC_50_ was calculated. Experiments were done in triplicates, and the mean of RFU/OD was plotted and used for the calculations of the maximum slope.

To test if the active compounds also inhibited QS in other bacterial species, the newly discovered compounds with QS inhibitor activity were tested on *P. aeruginosa*. GFP reporters for each of the effector protein pathways, Las, Rhl, and Pqs were used to test for QS inhibition in the *P. aeruginosa* strain PAO1. In addition, a PAO1-GFP strain was used as a control to test if the compounds were specific QS inhibitors or merely affected GFP or bacterial growth. GFP values were normalized to the growth of the bacteria, and the IC_50_ was determined based on the slope of the curves at different concentrations of the compound (Figure 5). Various compounds showed a clear, concentration-dependent reduction of the slope in one or more PAO1 QS reporters (Figure 5, Supplementary Figure 3). However, not all compounds with QS inhibitory activity in *C. violaceum* inhibited one or more QS pathways in PAO1. For most compounds, much higher concentrations were needed to inhibit QS in PAO1 than in *C. violaceum*. Overall, the compounds had the strongest effect on the *pqsA-*reporter and the least effect or no effect on the *rhlA*-GFP reporter.

An interesting difference between *C. violaceum* and PAO1 was also observed among the family of gregatins. The most promising compounds in *C. violaceum* were desmethyl-gregatin A and cyclogregatin. However, in PAO1, gregatin A and gregatin D seem to be the most potent QS inhibitors and they affected all the three pathways (Table 3). Therefore, gregatin A and gregatin D were selected for further analysis.

**Table 3:** QS inhibitor activity of selected compounds in *P. aeruginosa* (PAO1) reporters.

**Compound**	**IC_**50**_ lasB-GFP**	**IC_**50**_ rhlA-GFP**	**IC_**50**_ pqsA-GFP**	**IC_**50**_ PAO1-GFP**
Indole-3-acetic acid	Not active	Not active	>3125 μM	Not active
6-methyl salicylic acid	Not active	Not active	2809 μM	Not active
Rubrofusarin	Not active	>62,5 μM	17 μM	Not active
Citrinin	>2000 μM	Not active	1062 μM	Not active
Desmethyl Gregatin A	509 μM	Not active	130 μM	Not active
Gregatin A	228 μM	516 μM	203 μM	Not active
Gregatin D	282 μM	398 μM	294 μM	Not active
Cyclogregatin	>500 μM	Not active	>500 μM	Not active

*IC_50_ values of the different reporters were determined. Compounds were scored as “non-active” when it was not possible to plot a nonlinear regression curve because of lack of inhibitory activity. In case there was inhibitory activity, but 50% inhibition was not reached, the IC_50_ value was estimated to be higher than the highest concentrations tested. Higher concentrations could not be tested due to excessively high DMSO concentrations or precipitation of the compound*.

### Gregatins Increase Biofilm Formation in PAO1 QS Mutants

**Figure 6:**
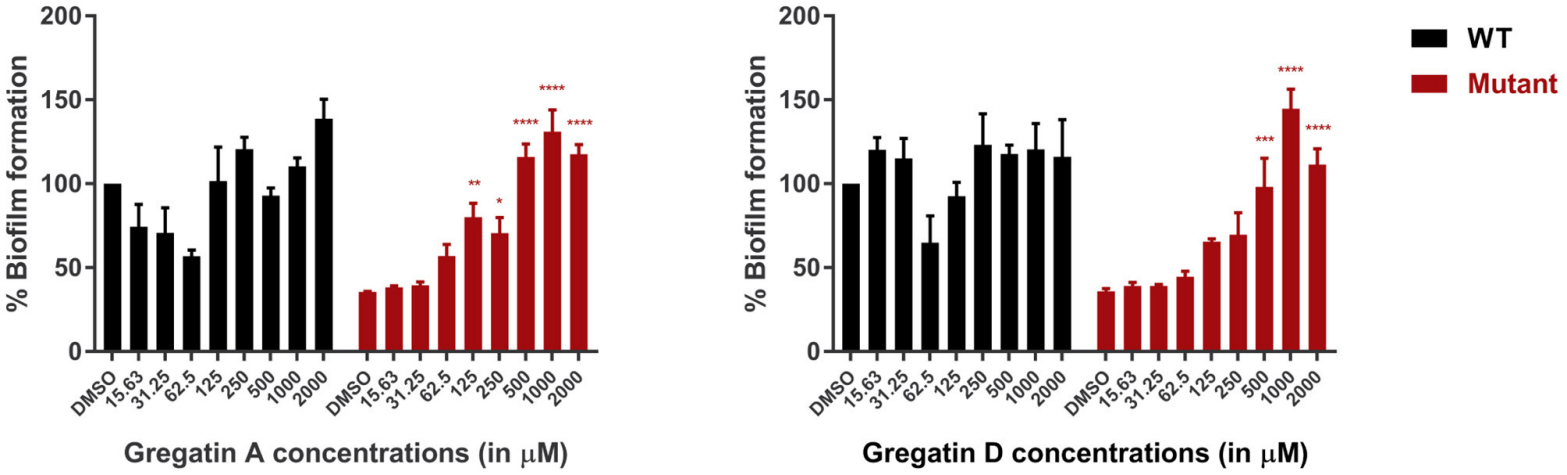
The effect of the gregatins on biofilm formation in *P. aeruginosa*. Biofilm formation was measured by crystal violet staining after treatment with gregatin A and gregatin D and normalized to DMSO treated. The effect on biofilm formation in both WT and QS mutants (Δ*lasI*/Δ*rhlI*) was measured. The mean of three experiments performed in triplicates was plotted. The error bars represent the SEM. A one-way ANOVA, corrected for multiple comparisons with Dunnett's test, was performed to determine statistical significance. Values are compared to DMSO-treated controls (*P < 0.05; **P < 0.005; ***P < 0.001; ****P < 0.0001).

Since QS is involved in the formation of biofilms, we hypothesized that inhibition of QS would inhibit biofilm formation. To test this, we measured the effect of gregatin A and gregatin D on the formation of biofilms in *P. aeruginosa* PAO1 using crystal violet staining. We tested the gregatins both on the formation of biofilm in WT bacteria and QS mutant (Δ*lasI-*Δ*rhlI*). Both gregatin A and gregatin D did not show a significant decrease or increase in biofilm formation in WT PAO1 (Figure 6). Interestingly, the biofilm formation was increased in QS mutants after treatment with gregatin A and gregatin D. Gregatin A appeared to be more potent than gregatin D. Both compounds showed maximum effects at 1,000 μM, at which concentration biofilm formation of the QS mutant exceeded that of WT (Figure 6). We conclude that gregatin A and gregatin D do not affect biofilm formation of PAO1 significantly, but may somehow affect biofilm formation in *P. aeruginosa* with impaired QS.

### Gregatins Alter the Expression Levels of Virulence Factors

**Figure 7:**
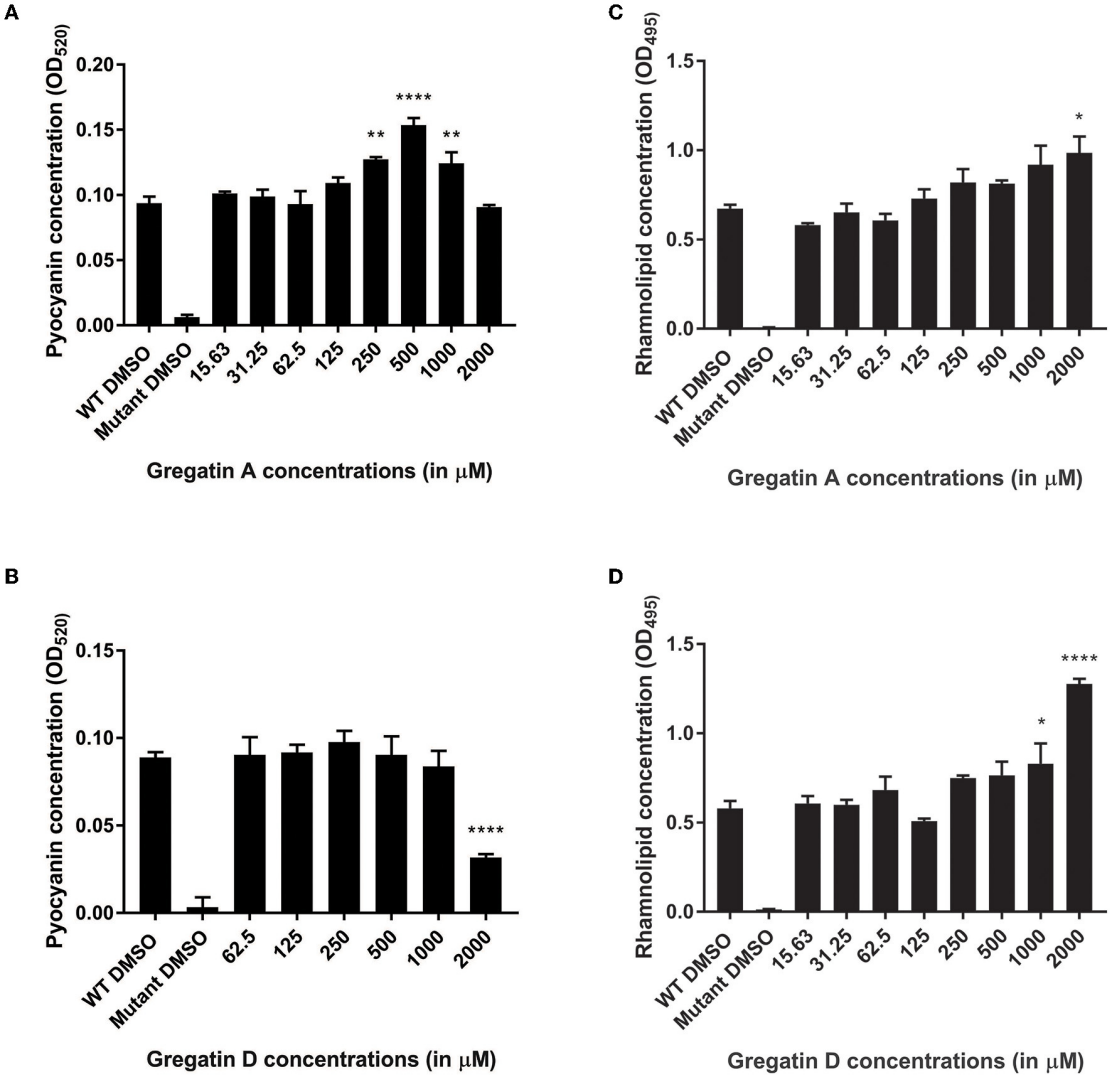
Distinct effects of gregatins on the production of virulence factors. The effect of gregatin A and gregatin D was tested on the production of the virulence factors **(A,B)** pyocyanin and **(C,D)** rhamnolipid. The mean of the experiment performed in triplicates was plotted. A one-way ANOVA, corrected for multiple comparisons with Dunnett's test, was performed to determine statistical significance. Values are compared to DMSO-treated controls (*P < 0.05; **P < 0.005; ****P < 0.0001).

Quorum sensing also regulates the production of virulence factors in *P. aeruginosa*. Therefore, we expected that the QS inhibitor activity of gregatin A and gregatin D would inhibit the production of virulence factors. To test this, we measured the relative levels of the virulence factors pyocyanin and rhamnolipids. As a control, the QS mutant was included, which produced more than 10-fold less virulence factor than WT PAO1 (Figure 7). Gregatin A treatment led to a dose-dependent increase in pyocyanin production in *P. aeruginosa* with an optimum at 500 μM (*p* < 0.0001) (Figure 7A). Gregatin D did not show an increase in pyocyanin expression, but a significant decrease (*p* < 0.0001) was observed at the highest concentration tested (2,000 μM) (Figure 7B). Gregatin A and gregatin D treatment increased rhamnolipid concentrations significantly only at high concentrations, that is, 2,000 and 1,000 μM, respectively, in stark contrast to diminished rhamnolipid production of the QS mutant (Figures 7C,D). Taken together, our data show different effects of gregatins on virulence factor production.

## Discussion

In this study, we found various active compounds in fungal filtrates that inhibited QS in *C. violaceum*. The identification of the known QS inhibitors (patulin and penicillic acid) showed that *C. violaceum* works well as a reporter in a high-throughput format. However, patulin and penicillic acid did not show a big difference between concentrations that affected QS inhibition and toxicity in bacteria, resulting in a small concentration range to evaluate QS inhibition without effects on viability. The newly identified QS inhibitors were more promising in this respect, and the family of gregatins was the most promising. While desmethyl-gregatin A and cyclogregatin showed the strongest inhibitory effect in *C. violaceum*, gregatin A and gregatin D showed a stronger effect in the opportunistic pathogen *P. aeruginosa*. Interestingly, although QS was inhibited in *P. aeruginosa*, gregatin A and gregatin D did not show inhibition of biofilm formation. Only gregatin D showed inhibition of pyocyanin production, whereas treatment with gregatin A led to an increase in pyocyanin synthesis. Treatment with both gregatins also led to an increase in rhamnolipid production.

While gregatin A has been identified as an anti-bacterial and anti-fungal agent (Anke et al., 1980), the function of other gregatins is not well-studied. Gregatins are a group of molecules with an alkylated furanone core (Burghart-Stoll and Brückner, 2012), which could explain the potency of gregatins as QS inhibitors. Furanones have been well-described as QS inhibitors, probably due to the high similarity of the ring structure to the lactone of the AHL autoinducers (Proctor et al., 2020). Therefore, the effect of the gregatins might be due to binding to the QS receptors.

Interestingly, although gregatins share a highly similar structure (Figure 4), the effect of different gregatins on *C. violaceum* and *P. aeruginosa* is distinct. Cyclogregatin and desmethyl-gregatin A showed stronger effects on *C. violaceum* than on *P. aeruginosa*, whereas gregatin A and gregatin D showed stronger effects on *P. aeruginosa* than on *C. violaceum*. This might be due to differences in the QS networks between these bacterial strains. Although the luxIR-type QS system in *C. violaceum* and *P. aeruginosa* shows resemblance, they do differ. The amino acid sequence of the luxR-type receptor of *C. violaceum* (CviR) shares 24 and 22% identity with LasR and RhlR from *P. aeruginosa*, respectively (Altschul et al., 1997, 2005). This might explain why the removal of a methyl group to get desmethyl-gregatin A alters the specificity of the compound. This also provides an opportunity for the chemical alteration of the compound to develop an optimized structure that shows strong QS inhibitor activity in multiple species.

It is interesting to note that various molecules with QS inhibitor activity on *C. violaceum* failed to show an effect on the opportunistic pathogen *P. aeruginosa*. This might be due to the robust, interconnected QS network in *P. aeruginosa. C. violaceum* has a single QS network (CviI/R) (Stauff et al., 2011), whereas *P. aeruginosa* uses three systems (LasI/R, RhlI/R, and PQS) (Lee and Zhang, 2014; Kostylev et al., 2019). These three systems regulate each other, but also show redundant effects. For example, in the absence of C4-HSL, RhlR is still activated *via* PqsE and regulates various downstream genes, including *rhlA* (Mukherjee et al., 2018). The robustness of the *P. aeruginosa* system might explain the high concentrations needed to inhibit QS compared to *C. violaceum*.

Another reason for the high concentrations needed or failure of QS inhibition in *P. aeruginosa* compared to *C. violaceum* might be the high intrinsic resistance of *P. aeruginosa*. All Gram-negative bacteria have a low permeability due to the structure of the outer membrane. However, *P. aeruginosa* shows a 12–100-fold lower permeability than *E. coli* due to the absence of general porins (Hancock, 1998; Chevalier et al., 2017). This low permeability makes it hard for compounds to cross the membrane and enter the bacteria. In addition, compounds that were able to cross the membrane are often targets for the efflux pumps, making it even harder to accumulate within *P. aeruginosa* (Dreier and Ruggerone, 2015).

Given the difference in effects on *C. violaceum* and *P. aeruginosa*, the question arises if *C. violaceum* is the best reporter bacterium to screen for QS inhibitors that are active in *P. aeruginosa*. The QS systems of *C. violaceum* and *P. aeruginosa* overlap partially. The CviI/R QS system of *C. violaceum* resembles the two AHL-dependent QS systems in *P. aeruginosa*, LasI/R, and RhlI/R. The PQS system of *P.aeruginosa* uses a distinct autoinducer (Williams and Cámara, 2009). High-throughput screens using *P. aeruginosa* reporters have been conducted before (Müh et al., 2006; Starkey et al., 2014; Kang et al., 2021). For instance, Starkey and colleagues fused the *pqsA* promoter to the *sacB* gene, resulting in a reporter bacterium that will only grow in a sucrose-supplemented medium when the PQS pathway is inhibited (Starkey et al., 2014). More specific screens for *P. aeruginosa* QS inhibitors using our fungal library or other sources of potential QS inhibitors may result in the identification of additional fungi with QS inhibitor activity in *P. aeruginosa*. Nevertheless, screening *C. violaceum* led to the identification of a range of QS inhibitors that are active against *P. aeruginosa* and may be active in other Gram-negative strains as well. Given the differential activities of the QS inhibitors that we identified in our screen toward different bacterial strains, it is important to verify the QS inhibitor activity in the bacterial strain of choice.

It is often reported that inhibition of QS leads to the inhibition of biofilm formation and the production of virulence factors (Duplantier et al., 2021). However, in this study, we only find an inhibitory activity of gregatin D on pyocyanin production, while it activates rhamnolipid synthesis and biofilm formation in QS mutants. Gregatin A also shows activation of both pyocyanin and rhamnolipid production. This might be due to the highly interconnected QS pathways in *P. aeruginosa*. For example, Welsh and colleagues found that QS inhibition leads to a decrease in pyocyanin production but an increase in rhamnolipids by agonistic binding to the Rhl receptor (Welsh et al., 2015). In addition, molecules specific to a single QS receptor do not show big effects on virulence factor production, and the effect is strongly dependent on the nutrient composition of the medium (Welsh and Blackwell, 2016). Smith and colleagues describe various QS inhibitors in *P. aeruginosa* that do not show an effect on biofilm formation nor on pyocyanin production (Smith et al., 2003a,b). They describe strong inhibition with Rhl inhibitors, whereas inhibitors specific to the Las pathway do not have a downstream effect. The effect of the QS inhibitors is weak on the Rhl pathway, which may explain the lack of downstream effects. Moreover, both biofilm formation and virulence factor production are also controlled by various other pathways and molecules (Bartell et al., 2017; Jakobsen et al., 2017; Huang et al., 2019; Perinbam et al., 2020). Transcriptomic analysis of a variety of QS inhibitors shows a wide variation in the number of genes affected by the QS inhibitors. This is probably due to the exact target of the inhibitor and its position in the hierarchy (Jakobsen et al., 2013). Therefore, to explain the contradictory results, it is important to identify the exact target of the gregatins.

Although we identified many fungal secondary metabolites that play a role in QS, there are still many more to be found. To find QS inhibitors, bacteria need to be treated at a specific concentration range. Low concentrations show no QS inhibitory activity, whereas high concentrations might be toxic. The fungi in the library were grown in a standard growth condition. Altering the growth conditions may lead to higher production of the active compound or synthesis of other metabolites (Bode et al., 2002). Hence, fungi that did not show activity in the current study might produce interesting compounds when grown under different conditions.

In conclusion, this study shows that fungi are a highly potent source of novel bioactive compounds. Through screening of 10,207 fungal filtrates, we found a diverse array of compounds that showed an inhibitory effect on QS, both in *C. violaceum* and in *P. aeruginosa*. Interestingly, we also found that QS inhibition in *P. aeruginosa* does not necessarily lead to a decrease in biofilm formation and production of virulence factors. It is important to find out more about the mechanism of action of gregatins, which may allow optimization of their structure to increase the potential of this family of compounds as QS inhibitors to ultimately combat antimicrobial resistance.

## Data Availability Statement

The raw data supporting the conclusions of this article will be made available by the authors, without undue reservation.

## Author Contributions

WB and JHe conceived and designed the study and wrote the first draft of the manuscript. JHo performed experiments. All authors contributed to manuscript revision, read, and approved the submitted version.

## Funding

This work was funded in part by a KNAW Research Fund grant.

## Conflict of Interest

The authors declare that the research was conducted in the absence of any commercial or financial relationships that could be construed as a potential conflict of interest.

## Publisher's Note

All claims expressed in this article are solely those of the authors and do not necessarily represent those of their affiliated organizations, or those of the publisher, the editors and the reviewers. Any product that may be evaluated in this article, or claim that may be made by its manufacturer, is not guaranteed or endorsed by the publisher.
