# Supplementary material for: Gregatins, a Group of Related Fungal Secondary Metabolites, Inhibit Aspects of Quorum Sensing in Gram-Negative Bacteria
Source: Front Microbiol. 2022 Jul 5;13:934235. doi: 10.3389/fmicb.2022.934235 (PMC9296082; doi:10.3389/fmicb.2022.934235)
Supplement: Supplementary file 1 [file Data_Sheet_1.PDF]

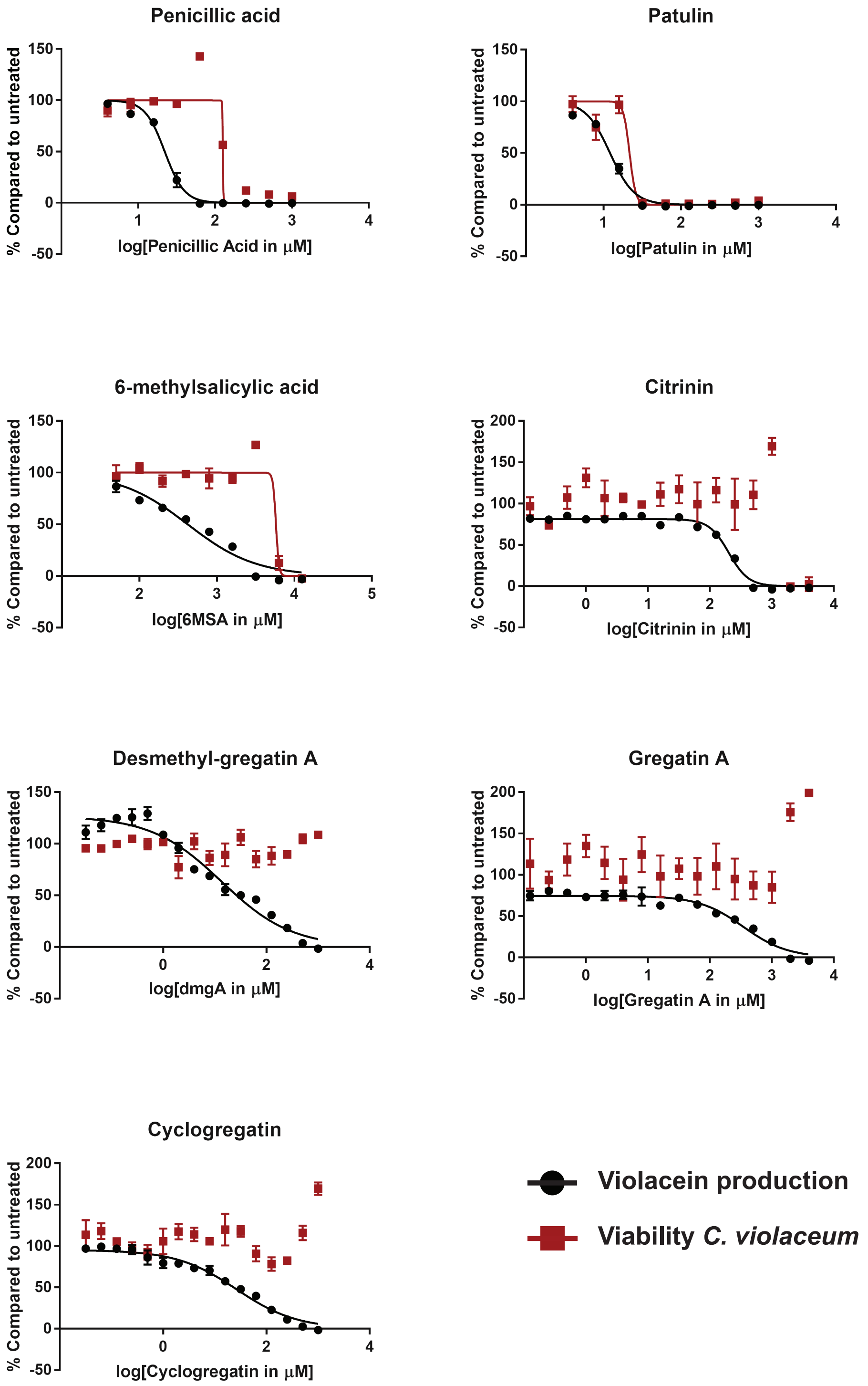

Supplementary Figure 1. Graphs of the compounds tested on the reporter *C. violaceum* of which the IC_{50} values were calculated (Table 2). Experiments were done in triplicate. Error bars represent SEM.

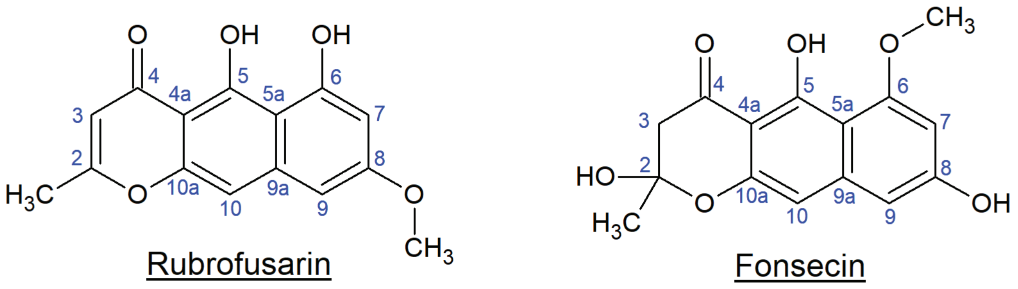

Position	Rubrofusarin		Fonsecin	
	^{1}H	^{13}C	^{1}H	^{13}C
2-CH_3	2,37 (3H, s, *J* =0.5)	20.5	1,6 (3H, s)	28.07
2		168.9		100.5
3	6,15 (1H, d, *J* =0.7)	107.0	2,74 (1H, d)<br>3,18 (1H, d, *J* =16.8)	48,0
4		184.1		198.1
4a		103.3		104.0
5		160.5	14,2 (1H, s)	164.7
5a		102.9		105.6
6		161.0		161.2
6-CH_3O			3,84 (3H, s, *J* =6.9)	56.1
7	6,42 (1H, s, *J* =2.7)	97.9	6,30 (1H, d, *J* =2.1)	97.0
8		161.8		162.8
8-CH_3O	3,86 (3H, s, *J* =5.4)	56.2		
9	, J=2.0H	101.5	6,46 (1H, d, *J* =2.1)	102.0
9a		141.4		143.4
10	7,03 (1H, s)	100.3	6,42 (1H, s, *J* =2.7)	101.5
10a		153.0		153.9

Supplementary Figure 2. NMR data of the active fraction from *Aspergillus carbonarius* suggesting a combination of fonsecin and rubrofusarin.

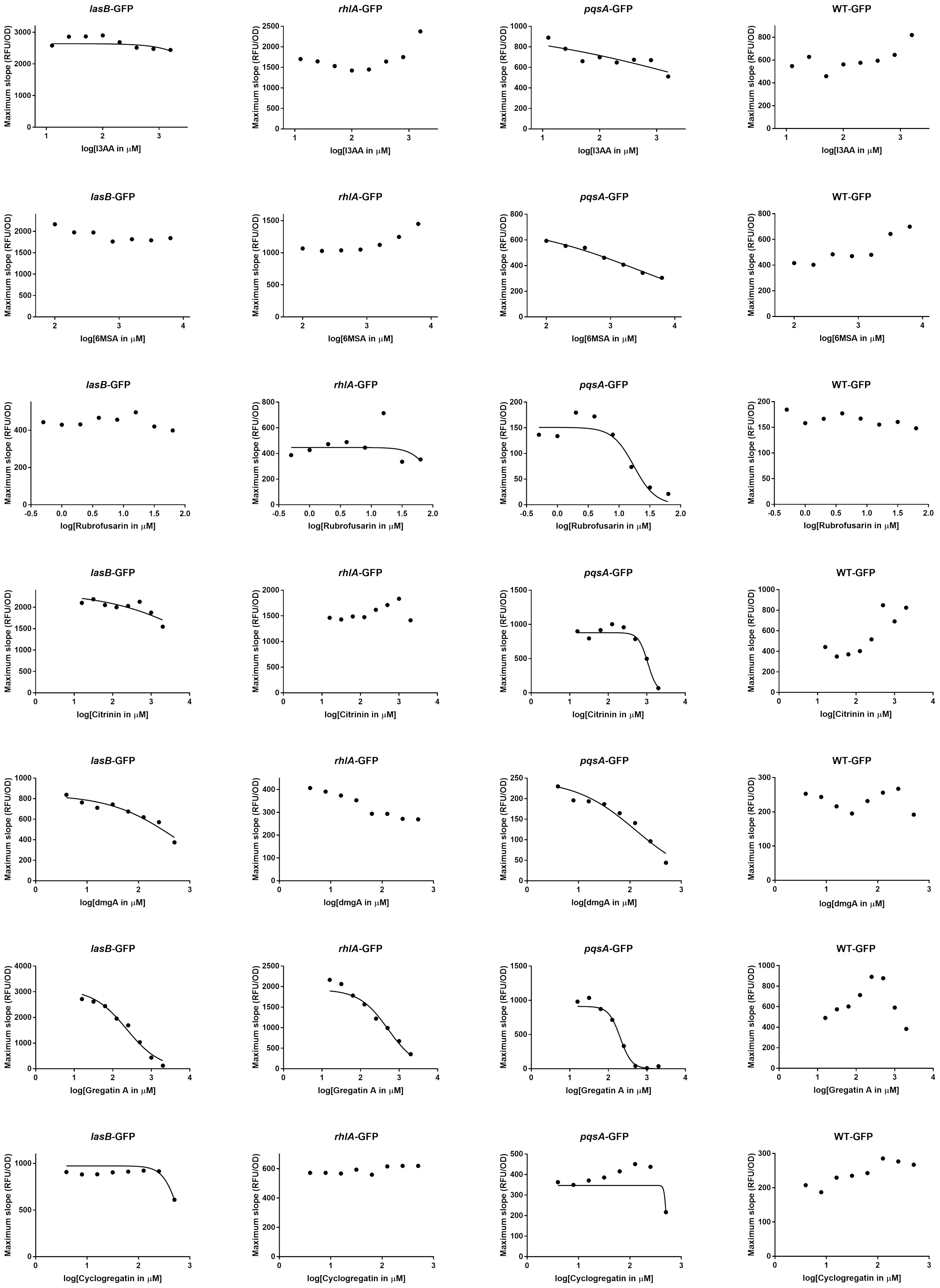

Supplementary Figure 3. QS inhibitory activity of the other compounds on *P. aerugonisa* strain PAO1. The plots represent the maximum slopes used to calculate the IC_{50} values. If no line is drawn, it was not possible to perform a non-linear regression analysis. Experiments were done in triplicate, the mean of RFU/OD is plotted and used for the calculations of the maximum slope.
